# Donor-Specific Blood Transfusion Induces a Transfusion-Related Early Protective Effect in Murine Lung Transplantation

**DOI:** 10.3389/ti.2026.15409

**Published:** 2026-04-30

**Authors:** Xin Jin, Charlotte Hooft, Balin Özsoy, Jan Van Slambrouck, Janne Kaes, Cedric Vanluyten, Annalisa Barbarossa, Marianne S. Carlon, Greetje Vande Velde, Mélanie Guyot, Karen Moermans, Steve Stegen, Robin Vos, Bart Vanaudenaerde, Jacques Pirenne, Laurens J. Ceulemans

**Affiliations:** 1 Lab of Respiratory Diseases and Thoracic Surgery, Department of Chronic Diseases, Metabolism (CHROMETA), KU Leuven, Leuven, Belgium; 2 Department of Thoracic Surgery, UZ Leuven, Leuven, Belgium; 3 Department of Imaging and Pathology, KU Leuven, Leuven, Belgium; 4 Laboratory of Angiogenesis and Vascular Metabolism, Department of Oncology, VIB-KU Leuven, Leuven, Belgium; 5 Clinical and Experimental Endocrinology, Department of Chronic Diseases, Metabolism (CHROMETA), KU Leuven, Leuven, Belgium; 6 Department of Respiratory Diseases, UZ Leuven, Leuven, Belgium; 7 Department of Abdominal Transplantation, UZ Leuven, Leuven, Belgium; 8 Lab of Abdominal Transplantation, Department of Microbiology, Immunology and Transplantation, KU Leuven, Leuven, Belgium

**Keywords:** allograft rejection, donor-specific blood transfusion, immune regulation, immunosuppression, lung transplantation

## Abstract

Lung transplantation (LTx) remains limited by high immunogenicity and chronic rejection, yet the application of donor-specific blood transfusion (DSBT) in LTx has not been fully investigated. We established a murine orthotopic LTx model (BALB/c to C57BL/6N) to evaluate the safety and efficacy of DSBT administered 24 h prior to transplantation. Pre-transplant transfusion was well-tolerated showing no evidence of Transfusion-Related Acute Lung Injury (TRALI) or volume-overload injury. Radiographic analysis at POD 7 demonstrated that DSBT-treated grafts maintained significantly higher aerated lung volume compared to non-transfused controls. Flow cytometric analysis at the same time point revealed that these grafts were preferentially infiltrated by recipient-derived monocytes and type 2 conventional dendritic cells (cDC2s), while lymphoid cell counts remained comparable across groups in both the lung and spleen, indicating no systemic immune depletion. By POD 35, however, histological analysis revealed that the lung grafts were extensively destroyed by severe rejection. These findings demonstrate that a 24-h pre-transplant DSBT window improves early graft patency and modulating the localized myeloid landscape in a murine model. We conclude that DSBT serves as a safe and effective induction strategy to mitigate early inflammatory consolidation, providing a predictable temporal window for secondary immunomodulatory interventions in lung transplantation.

## Graphical Abstract

**Figure:**
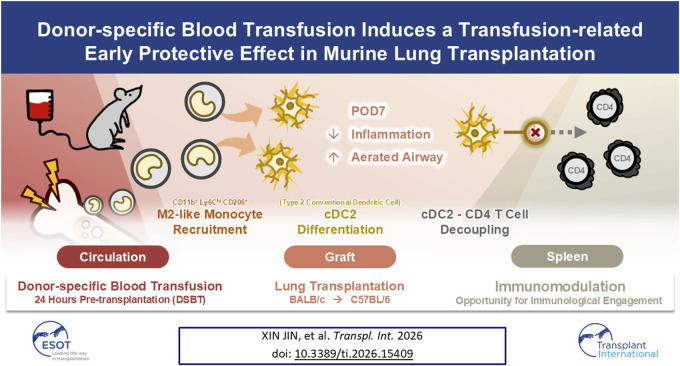

## Introduction

According to the latest report of the International Society for Heart and Lung Transplantation (ISHLT), the 5-year survival rate for lung transplantation is approximately 59%, which is the lowest among all solid organ transplants [1, 2]. Lung transplant patients require lifelong immunosuppression to suppress the intense immune response elicited by major histocompatibility complex (MHC) incompatibility, which is significantly more intense in the lung than in the other solid organs. Despite being the ultimate treatment for end-stage pulmonary disease, its long-term success is hindered by the fragile balance between the necessity for profound immunosuppression carrying risks of nephrotoxicity, malignancy, and opportunistic infection versus the persistent threat of chronic allograft rejection [3, 4]. The future of lung transplantation depends on strategies that expand the therapeutic window by promoting graft acceptance and simultaneously limiting the need for profound immunosuppression and its associated complications.

Donor-specific blood transfusion (DSBT), the infusion of fresh donor whole blood containing all blood cell types and plasma proteins into the recipient prior to transplantation, has been reported to improve graft acceptance and potentially induce a tolerogenic state [5]. Since its first application in canine allogeneic kidney transplantation in 1964 and in human kidney transplantation in 1980, multiple studies including our Leuven Immunomodulatory Protocol in intestinal transplantation have demonstrated that DSBT can prolong graft survival and reduce the required dosage of postoperative immunosuppressants [6–9]. However, the application of DSBT remains unexplored in the context of LTx, and its underlying immunomodulatory mechanisms in the pulmonary environment remain largely elusive.

In this proof-of concept study, we first evaluated the safety of DSBT by investigating its impact on pulmonary integrity and systemic hematological profiles in a murine model. Furthermore, we established the first murine orthotopic LTx model of DSBT preconditioning to analyze its effects on graft and immune cell dynamics, providing a novel framework for understanding the regulation of lung allograft rejection.

## Materials and Methods

### Blood Transfusion and Orthotopic Left LTx Mouse Model (Figure 1)

The project was approved by the Ethical Committee for Animal Research at KU Leuven (P015/2024). Eight-to 12-week-old BALB/c (H2K^d^) and C57BL/6N (H2K^b^) mice were purchased from Janvier Labs (Le Genest-Saint-Isle, France). All animals were housed in a conventional facility with individually ventilated cages and had access to standard chow and water *ad libitum*.

**FIGURE 1:**
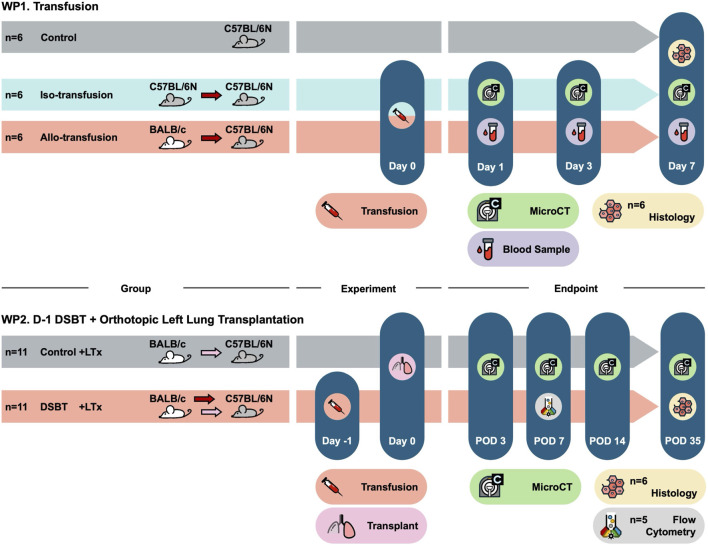
Study design. WP1: safety of 0.2 mL blood transfusion via tail vein in mouse model; WP2: efficacy of DSBT in orthotopic left lung transplant mouse model. DSBT: donor-specific blood transfusion; LTx: lung transplantation; POD: post-operative day.

WP1. Transfusion:

Donor: C57BL/6N (iso-transfusion, n = 6)
or BALB/c (allo-transfusion, n = 6)
Recipient: C57BL/6N
Control: C57BL/6N non-transfused mice (n = 6)

Donor mice were anesthetized using 5.0% isoflurane (Iso-Vet, Piramal Critical Care, the Netherlands) in room air, intubated, and maintained under anesthesia with 2.0% isoflurane. Ventilation was provided using a microventilator (UNO microventilator, the Netherlands) at a respiratory rate of 120 breaths per minute with a tidal volume of 275–300 μL. Inferior vena cava was exposed by laparotomy, and 0.8–1.0 mL of whole blood (for three recipients) was collected into a heparinized centrifuge tube which was stored at 4 °C fridge until transfusion.

Recipient transfusion mice were anesthetized with 3.0% isoflurane in room air. A total of 0.20 mL of whole blood was withdrawn into an insulin syringe (0.3 mL U-100 30 G, BD, USA) and administered via the lateral tail vein. The interval between blood collection and transfusion did not exceed 30 min.

All mice that received transfusion only were euthanized on day 7, and the left lung was procured for histological analysis.

WP2. D-1 DSBT + Orthotopic Left LTx:

Experiments were randomized.
Donor: BALB/c (blood and lung)
Recipient: C57BL/6N with DSBT 24 h prior to LTx (D-1 DSBT + LTx, n = 11)
Control: C57BL/6N non-transfused mice (Control + LTx, n = 11)

The surgical procedure for murine orthotopic left LTx was performed as previously described in our review on murine LTx [10]. Briefly, both donor and recipient mice were anesthetized with isoflurane and connected to a ventilator.

Procurement: A total of 100 μL of heparin (LEO Pharma, Denmark) was injected into the inferior vena cava, followed by perfusion of the heart–lung bloc with 2–3 mL of sterile 4 °C saline via the pulmonary artery. The left pulmonary artery, bronchus, and pulmonary vein were carefully dissected and cuffed using 24-, 20-, and 22-gauge cuffs, respectively. The donor lung was stored at 4 °C fridge until implantation.

Transplantation: In the recipient, the left hilum was exposed, and the vessels were occluded using 9-0 slip knot sutures. The anastomoses were performed in the sequence of bronchus–vein–artery, deviating from the previously reported artery–bronchus–vein sequence, to optimize surgical outcomes. The second warm ischemic time (removal of the graft from cold storage to reperfusion) should be less than 20 min to maintain a low-inflammatory baseline and minimize confounding effects from ischemia-reperfusion injury. The native left lung was removed, and the thoracic incision was closed using 6-0 or 7-0 running sutures.

Postoperative care: For analgesia, buprenorphine (0.1 mg/kg Vetergesic, Ecuphar, Netherlands) was administered subcutaneously twice daily until postoperative day (POD) 3. To prevent graft rejection, recipients received subcutaneous injections of methylprednisolone (1.6 mg/kg; SoluMedrol, Pfizer, Belgium) and cyclosporine (10 mg/kg; Sandimmun, Novartis, Belgium) once daily starting from POD 1. All recipients were euthanized on POD 35, and the left lung grafts were procured for histological analysis.

### Endpoints

#### Clinical Endpoints

During the follow-up period, all animals were monitored and weighed daily. Euthanasia was performed if any of the following endpoints were met: 1) irreversible body weight loss exceeding 20%; 2) complete loss of appetite or inability to drink; 3) severe respiratory or circulatory distress, including gasping, edema, or cyanosis of the extremities; or 4) abnormal behavior or movement, such as hyperactivity, lethargy, or abnormal muscle tone.

#### Blood Sample Analysis (WP1)

The complete blood count by facial vein phlebotomy collection was conducted using hematology analyzer (Element HT5, Heska, US) according to the machine manual on Day 1, 3 and 7 after transfusion.

#### *In Vivo* microCT Imaging (WP1&2)

For transfusion-only recipients, *in vivo* microCT scans were performed on Day 1, 3, and 7.

For transplant recipients, scans were conducted on POD 3, 7, 14, and 35. Recipients presenting with complete consolidation of the left lung graft within the first 3 days, suggestive for technical failure [primary graft dysfunction (PGD) or severe inflammation] were excluded and replaced by new animals.

During scanning, animals were anesthetized with 2.0% isoflurane in pure oxygen and positioned supine. Imaging was performed with SkyScan 1278 *in vivo* microCT scanner (Bruker, Belgium). Scanning and reconstruction (NRecon, v1.7.0.4; CTan software, v1.16.8.0, Bruker, Belgium) parameters were set according to our protocol [11]. Pixel with a gray value of 0–80 is defined as aerated lung tissue. The number of voxels and mean gray value were further calibrated to volume in mL and density in Hounsfield Units (HU).

#### Histological Analysis (WP1&2, n = 6)

Histological analysis was performed on POD 35 according to our established protocol [11]. At the time of sacrifice, lungs were ventilated *in vivo* and perfused with saline via the pulmonary artery, followed by perfusion with 4% paraformaldehyde (PFA). The trachea was ligated under full inflation, and the lungs were fixed in 4% PFA at 4 °C for 12–24 h.

Lung grafts were processed into 4 μm formalin-fixed paraffin-embedded sections and stained with hematoxylin and eosin [WP1, n = 6; WP2, n = 6 (Leica, Germany), H&E] and Masson’s trichrome kit [WP2, n = 6 (Sigma-Aldrich, US)] following the manufacturer’s instructions. All sections were blindly evaluated by experienced pulmonary pathologists according to ISHLT grading system for cellular rejection [12].

The percentage of collagen deposition in lung sections stained with Masson’s trichrome was quantified using QuPath software (v0.6.0) with the Pixel Classifier Tool.

#### Flow Cytometry Analysis (WP2, n = 5)

Flow cytometry analysis was performed on POD 7 according to our established protocol [13]. At the time of euthanasia, the recipients were anesthetized via an intraperitoneal injection of xylazine (100 mg/kg; Xyl-M, VMD, Belgium) and ketamine (10 mg/kg; Nimatek, Dechra, Belgium). To distinguish marginated circulatory cells from tissue-resident cells, 50 μL of CD45.2 antibody solution (0.2 mg/mL in PBS) was injected into the inferior vena cava. Five minutes later, the lung graft and spleen were harvested and processed into single-cell suspension. Absolute cell numbers in the lung and spleen were quantified using a flow cytometer (BD LSRFortessa™; Water Biosciences, US). The gating strategy (Supplementary Material 1) included total viable cells (Live/Dead negative), myeloid cells, lymphoid cells, and cell chimerism (H2K^b/d^). Data were analyzed using FlowJo software (v10.6.0, Waters Biosciences, US).

### Statistical Analysis

Data were analyzed using GraphPad statistical software (v10.2.1, Prism, US) and SPSS (v20.0.0, IBM, US). Mixed-effects model with multiple comparisons (mean ± SD, for longitudinal data), Mann-Whitney U tests [Median (Min-Max), for non-normal distribution data], Chi-square test (for categorical variables) and Kaplan-Meier analysis (for graft survival) were used to compare intergroup differences. A P-value <0.05 was considered significant. In cases where non-parametric analysis failed to reach significance but suggested a clear distributional trend, we applied bootstrapping techniques (10,000 iterations) to re-evaluate the comparative differences with increased statistical power.

## Results

### Transfusion

#### Mice Remained Clinically Stable After Transfusion (Figure 2A)

Before transfusion, the weight of iso-transfusion mice was 26.58 ± 4.59 g, and that of allo-mice was 23.02 ± 3.03 g (P = 0.180). On Day 7, the weight of iso-mice increased by 4.0%–27.43 ± 3.55 g, and the weight of the allo-mice increased by 2.3%–23.50 ± 2.82 g (P = 0.093). All mice remained clinically stable during observation with no development of organ dysfunction or reaching humane endpoints.

**FIGURE 2:**
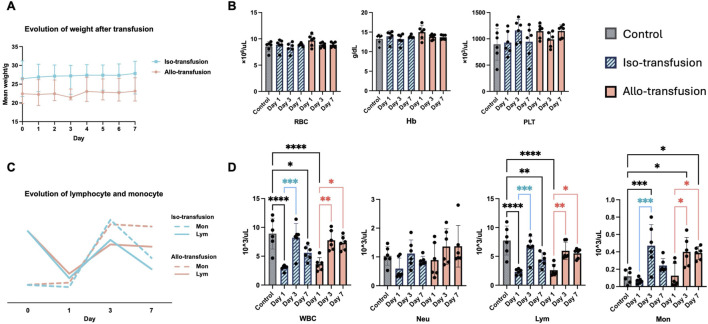
Mice remained clinically stable after transfusion, and no transfusion-related hemolysis occurred. A transfusion-related effect was observed since Day 1. **(A)** recipient weight change after transfusion which slightly increased in both Iso- (4.0%) and Allo-transfusion (2.3%) group; **(B)** the stable level of hemoglobin and red blood cell and platelet count in evidence of no occurrence of hemolysis or coagulation disorder; **(C)** visualization of the trend of lymphocyte and monocyte; **(D)** the significant decrease of white blood cell count (and lymphocyte) on Day 1 and the increase (white blood cell, lymphocyte and monocyte) on Day 3. Moreover, the lymphocyte and monocyte count showed a different evolution pattern in Iso-vs. Allo-transfusion on Day 7. *P < 0.05, **P < 0.01, ***P < 0.001, and ****P < 0.0001. Hb: hemoglobin; Lym: lymphocyte; Mon: monocyte; Neu: neutrophil; PLT: platelet; RBC: red blood cell; WBC: white blood cell.

#### No Transfusion-Related Hemolysis Occurred and a Transfusion Effect on White Blood Cells was Observed (Figures 2B–D)

Compared with the control group, there were no significant changes in red blood cell, platelet counts or hemoglobin level during the 7-day follow-up in either group.

White blood cells count decreased significantly on Day 1 from 8.922 × 10^3^/μL to 2.990 × 10^3^/μL in the iso-transfusion group (P < 0.001) and to 3.625 × 10^3^/μL in the allo-group (P < 0.001). By Day 3, white blood cells count recovered to 8.200 × 10^3^/μL in the iso-group (P > 0.999) and to 7.752 × 10^3^/μL in the allo-group (P = 0.986). On Day 7, white blood cells count in the allo-group remained comparable to baseline (7.298 × 10^3^/μL, P = 0.834), whereas a significant decline was observed in the iso-group (5.595 × 10^3^/μL, P = 0.030).

Lymphocytes count exhibited a similar trend, with a significant decrease on Day 1 (control: 7.738 × 10^3^/μL; iso-: 2.317 × 10^3^/μL, P *<* 0.001; allo-: 2.602 × 10^3^/μL, P *<* 0.001), a recovery on Day 3 (iso-: 6.573 × 10^3^/μL, P *=* 0.953; allo-: 5.963 × 10^3^/μL, P *=* 0.512), and a divergent trend on Day 7 (iso-: 4.458 × 10^3^/μL, P *=* 0.008; allo-: 5.522 × 10^3^/μL, P *=* 0.196).

Monocytes count did not change significantly on Day 1 (control: 0.118 × 10^3^/μL; iso-: 0.067 × 10^3^/μL, P *>* 0.999; allo-: 0.125 × 10^3^/μL, P *>* 0.999) but increased on Day 3 (iso-: 0.470 × 10^3^/μL, P *=* 0.001; allo-: 0.400 × 10^3^/μL, P *=* 0.013). This increase persisted until Day 7 in the allo-group (0.387 × 10^3^/μL, P *=* 0.021) but returned to baseline in the iso-group (0.243 × 10^3^/μL, P *=* 0.843).

Neutrophil counts showed a pattern similar to monocyte, but this did not reach statistical significance at any time point.

#### No Transfusion-Related Acute Lung Injury Occurred (Figure 3)

**FIGURE 3:**
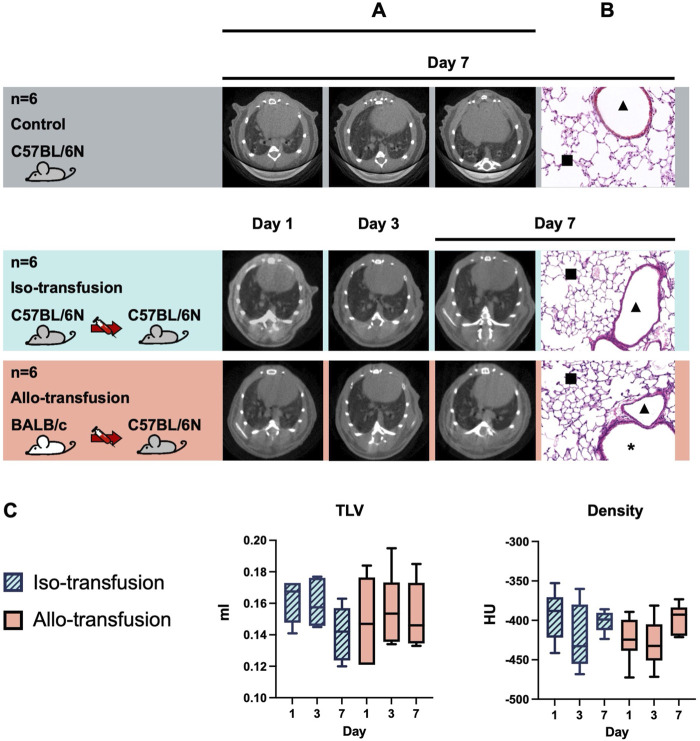
No transfusion-related acute lung injury occurred after DSBT. Representative pictures of **(A)** *in vivo* microCT and **(B)** hematoxylin and eosin (H&E) stain, magnification ×20, [**▲**] indicates vessels, [*] indicates airways, [■] indicates parenchyma; **(C)** evolution of total lung volume (TLV) and mean density of left lung.

MicroCT imaging revealed normal lung morphology at all time points in both the iso-transfusion and allo-transfusion groups (Figure 3A). No evidence of transfusion-related acute lung injury (TRALI) was observed in either group as lung grafts cellular rejection all graded as A0 (Figure 3B). There were no signs of consolidation indicative of edema, pneumonia, or pleural effusion, as reflected by stable left lung volumes (iso-vs. allo-: Day 1: 0.162 ± 0.012 mL vs. 0.149 ± 0.024 mL; Day 3: 0.160 ± 0.013 mL vs. 0.156 ± 0.022 mL; Day 7: 0.141 ± 0.016 mL vs. 0.152 ± 0.019 mL; all P *>* 0.05) and density (iso-vs. allo-: Day 1: -393.9 ± 28.6 HU vs. −423.4 ± 26.0 HU; Day 3: -421.9 ± 37.9 HU vs. −429.1 ± 28.4 HU; Day 7: -401.3 ± 12.2 HU vs. −397.7 ± 17.2 HU; all P *>* 0.05) throughout the follow-up period (Figure 3C).

### D-1 DSBT + Orthotopic Left LTx

#### The Protective Effect of DSBT Was Biphasic as the Most Obvious on POD 7 but not Observed by POD35 (Figures 4, 5)

The median interval between DSBT and LTx was 26.1 (24.2–27.9) hours. The cold ischemia time, defined as the duration from pulmonary artery flush to retrieval from refrigeration, was 69 (48–100) minutes in the control group and 55 (51–61) minutes in the DSBT group. Warm ischemia time, was similar between groups: 16.5 (10–20) minutes in the control group and 16.5 (14–20) minutes in the DSBT group (P *=* 0.842). The total surgical duration was comparable in both group [control: 65 (51–74) vs. DSBT: 64 (55–70) minutes (P *=* 0.732)].

**FIGURE 4:**
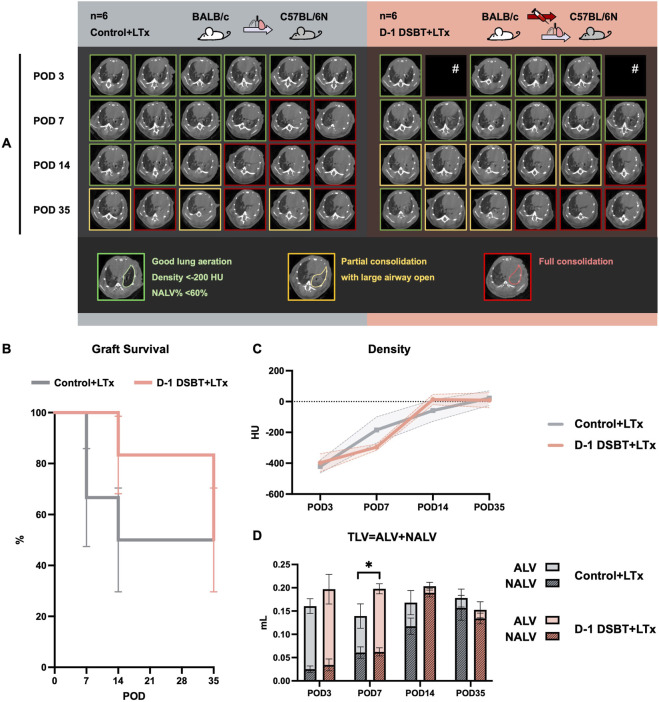
The evolution of the density and volume of left lung graft on condition of no acute complication on POD 3^#^. The protective effect of DSBT was the most obvious on POD 7 but not observed by POD35 in mouse lung transplantation. **(A)** representative pictures of *in vivo* microCT image of each recipient on POD 7, 14 and 35, image with clear lung markings on the left side was labelled with green, partial consolidation with yellow and full consolidation with red. **(B)** (functional) graft survival if no full consolidation image on CT; evolution of left lung graft’s **(C)** mean density and **(D)** volume. *P < 0.05. ALV: aerated lung volume; DSBT: donor-specific blood transfusion; NALV: non-aerated lung volume, TLV: total lung volume; POD: post-operative day. ^#^POD3 images of two mice of DSBT group was missing due to a mechanical issue. They were still included in the study because of their stable status, no abnormal activity and good lung aeration images on POD7 (they would not recover if full consolidation happened on POD3).

**FIGURE 5:**
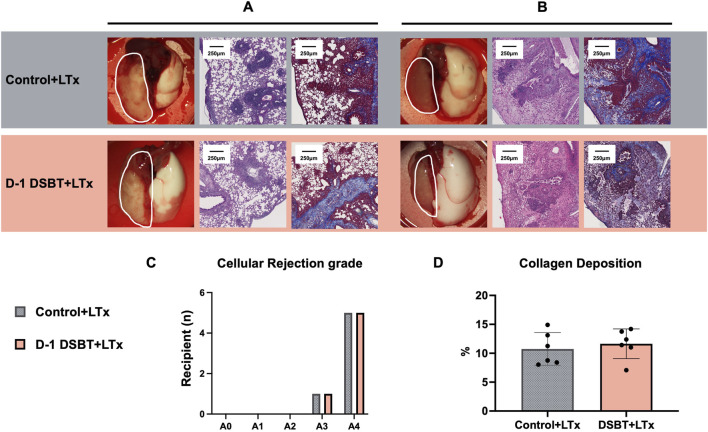
Histological analysis of the left lung graft on POD 35 after lung transplantation. The protective effect of DSBT was not observed by POD 35. Representative pictures of macroscopic view (left, labelled with white frame), hematoxylin and eosin staining (middle) and Masson’s trichrome (right, collagen dyed in blue) of **(A)** moderate rejection (cellular rejection grade A3) and **(B)** severe rejection in left lung graft (cellular rejection grade A4); **(C)** cellular rejection grade of left lung graft; **(D)** collagen deposition in left lung graft measured by QuPath.

Three surviving recipients in each group were excluded due to complete consolidation of the left lung graft observed by microCT on POD 3 and were replaced by no-consolidated mice. No animals reached criteria for euthanasia during the 35-day follow-up period.

On POD 7, microCT scans (n = 6, Figure 4A) revealed complete graft consolidation in 33.3% of mice in the control group, whereas 100% of mice in the DSBT group showed well lung aeration (P *=* 0.455). By POD 14, the proportion of functional grafts remained higher in the DSBT group compared with the control group (83.3% vs. 50%, P *=* 0.546). On POD 35, 50% of the left lung grafts remained functional in both groups (Figure 4B, Kaplan-Meier curve P = 0.489).

Total left lung volume (TLV) is presented as side-by-side stacked bars, partitioned into aerated (ALV) and non-aerated (NALV) fractions in longitudinal assessment of microCT on POD 3, 7, 14, and 35 (Figures 4C,D). The DSBT group exhibited a significantly more stable TLV trajectory compared to the decline observed in the control group during the early post-operative phase (Interaction P = 0.024). Notably, at POD 7 and POD 14, DSBT-treated lungs maintained a higher proportion of functional ALV while effectively limiting the expansion of pathological NALV (Interaction P = 0.034).

Histological assessment (n = 6) on POD 35 showed comparable cellular rejection grades [control vs. DSBT: (A3 n = 1, A4 n = 5) vs. (A3 n = 1, A4 n = 5), P *=* 1.000; Figure 5C] and similar fibrosis scores (collagen deposition, control vs. DSBT: 10.01% vs. 11.91%, P *=* 0.699; Figure 5D).

#### Increased Infiltration of Pro-Reparative Monocyte-cDC2 Axis With Concomitant Suppression of Systemic Immune Activation on POD 7

**FIGURE 6:**
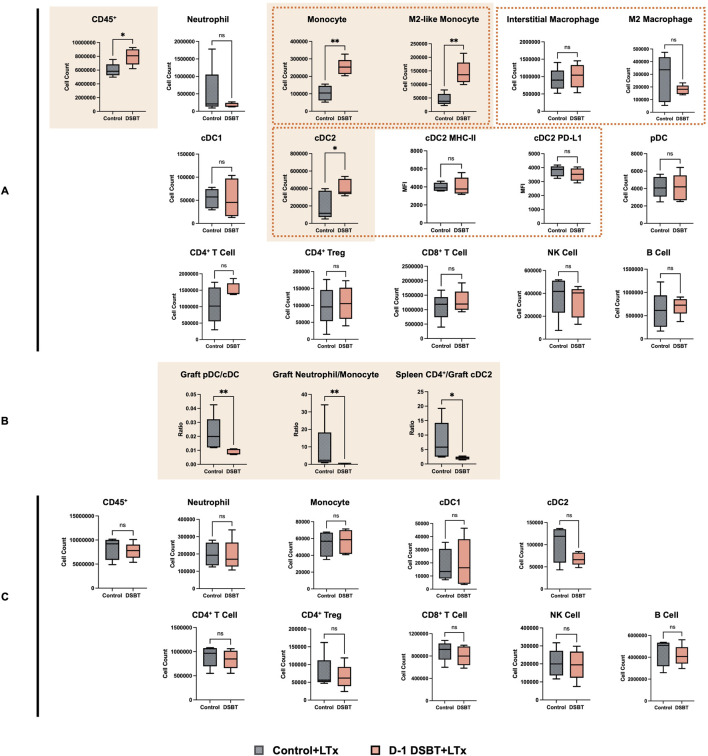
DSBT treatment recalibrated the intragraft myeloid landscape and induces functional immune decoupling by POD 7. Flow cytometric analysis was performed on left lung graft and recipient spleen on POD 7 (n = 5). **(A)** recalibration of the myeloid pool in lung grafts. DSBT treatment significantly expanded the recipient-derived CD11b^+^ Ly6C^hi^ Ly6G^−^ monocyte population, particularly the pro-reparative CD206^+^ M2-like subset. Despite this influx, the downstream interstitial macrophage pool remained stable, and increased infiltrating CD11b^+^ XCR1^-^ cDC2s remain functionally controlled without further upregulation of MHC-II or PD-L1 expression. **(B)** lineage bias in monocyte differentiation. Within the graft, infiltrating monocytes preferentially shifted toward a potentially immunoregulatory cDC2 fate rather than a pro-inflammatory pDC lineage (decreased pDC/cDC and NMR). **(C)** systemic immune quiescence in the spleen. The localized accumulation of cDC2s and M2-like monocytes in the graft did not translate into systemic CD4^+^ or CD8^+^ T cell activation *P < 0.05, **P < 0.01, ns: no significance. cDC: conventional dendritic cell; pDC: plasmacytoid dendritic cell; LTx: lung transplantation; MFI: mean fluorescent intensity; NK cell: natural killer cell; Treg: regulatory T cell; NMR: neutrophil-to-monocyte ratio.

Given that the most pronounced protective effect of DSBT was observed on POD 7, we performed a detailed flow cytometric analysis of lung grafts and recipient spleens (n = 5 per group) to explore the underlying mechanisms. In the DSBT group, the total count of intragraft recipient-derived CD45^+^ immune cells was significantly higher than in control group (8.08 × 10^6^ vs. 5.82 × 10^6^, P = 0.032, Figure 6A). This increase was primarily driven by a robust expansion of CD11b^+^ Ly6C^hi^ Ly6G^−^ monocytes (2.53 × 10^5^ vs. 1.05 × 10^5^, P = 0.008). Notably, these infiltrating monocytes were predominantly of the pro-reparative CD206^+^ M2-like subset (13.58 × 10^4^ vs. 3.80 × 10^4^, P = 0.008). Beyond monocyte expansion, we observed a distinct lineage bias in the DSBT group: infiltrating myeloid cells preferentially differentiated into potentially immunoregulatory cDCs, specifically CD11b+ XCR1-cDC2s (3.58 × 10^5^ vs. 1.15 × 10^5^, P = 0.048), rather than pro-inflammatory plasmacytoid dendritic cells (pDCs. Intragraft pDC/cDC ratio:0.008 vs. 0.020, P = 0.008, Figure 6B) [14]. Furthermore, the DSBT group exhibited a markedly lower neutrophil-to-monocyte ratio (NMR, 0.618 vs. 2.354, P = 0.008), suggesting a shifted inflammatory milieu that favors monocyte-mediated regulation over neutrophil-driven injury. Crucially, despite the increased graft cDC2 infiltration, the ratio of splenic CD4^+^ T cells-to-graft cDC2s was significantly reduced in the DSBT group (2.198 vs. 5.819, P = 0.032), indicating a functional cDC2-CD4^+^ T cell decoupling. No significant differences were observed in the absolute counts of other donor- or recipient-derived myeloid or lymphoid cell subsets (Figure 6C, Supplementary Material 2), underscoring the specificity of the DSBT-induced myeloid recalibration.

## Discussion

In this study, we established a novel preconditioning platform for murine LTx and identified a distinct biphasic immune response induced by DSBT, integrating the foundational DSBT principles of Fabre *et al.* with the surgical precision of Okazaki’s cuff-technique [15, 16]. Our data demonstrate that DSBT provides a transient period of graft protection characterized by preserved parenchymal aeration and a recalibrated myeloid landscape on POD 7. However, this effect eventually faded out by a rejection flare-up by POD 35. Rather than viewing the late failure merely as a therapeutic limitation, we propose that DSBT acts as a powerful spatiotemporal modulator that converts chaotic acute injury into a predictable “Window of Opportunity for Immunologic Engagement (WOFIE),” a conceptual framework to describe the essential engagement between the host immune system and the allograft required for tolerance induction [17].

From a translational perspective, the primary concern regarding DSBT is the potential induction of Transfusion-Related Acute Lung Injury (TRALI). According to the “two-hit” hypothesis, TRALI requires an initial priming of the host environment (first hit) followed by the activation of neutrophils by transfused components (second hit) [18]. In our model, the absence of evidence of diffuse alveolar damage suggests that DSBT, when administered 24 h prior to surgery, does not incite TRALI-like acute injury. This safety profile is likely attributed to the low-inflammatory threshold at the time of transfusion as prioritized in the Leuven Immunomodulatory Protocol [19, 20]. By bypassing systemic inflammatory priming during the transfusion window, DSBT appears to serve as a safe pre-conditioning intervention that favors homeostatic recalibration over hyperacute sensitization.

The 24-h interval between DSBT and LTx likely orchestrates the homing of DSBT donor cells to secondary lymphoid organs and a systemic recipient myeloid recalibration rooted in the concept of “trained immunity” [21]. As the primary biological filter for intravenous cargo, the spleen likely serves as the sensory hub for this process. While we observed quantitative stability in the splenic compartment (Figure 6C), its specialized microenvironment is evolutionarily optimized to process blood-borne antigens and initiate systemic tolerogenic signals. Recent evidence has identified a robust spleen-bone marrow axis. The splenic sensing of peripheral immunomodulation which could be the immunogenic components in DSBT in this study triggers the release of systemic mediators that migrate to the bone marrow to recalibrate hematopoietic stem and progenitor cells (HSPCs) [5, 22, 23]. This process likely biases the bone marrow output toward monocyte-dendritic cell progenitors (MDPs) over the pro-inflammatory granulocyte-monocyte progenitor (GMP) lineage [24, 25].

In contrast to the pro-inflammatory role of GMP-derived neutrophils inducing acute lung injury, MDP-derived cells appear predisposed toward a reparative and homeostatic phenotype following DSBT. This systemic recalibration manifests at POD 7 as a robust expansion of recipient-derived CD11b^+^ Ly6C^hi^ CD206^+^ M2-like monocytes (P = 0.008) and CD11b^+^ XCR1^-^ cDC2s (P = 0.048), coupled with a significant reduction in the intragraft NMR (P = 0.008). Consistent with the findings of Menezes *et al.*, who demonstrated that the intrinsic heterogeneity of Ly6C^hi^ monocytes dictates their developmental fate toward either dendritic cells or macrophages [26], our data suggest that DSBT effectively “primes” the monocyte pool toward a cDC2-lineage bias. This deliberate expansion of the pro-reparative monocyte pool, coupled with the numerical stability of mature macrophage populations (P = 0.548), suggests that DSBT does not merely suppress inflammation, but rather re-engineers the graft’s cellular architecture. Building on the “niche occupancy” theory, we propose that these primed monocytes, rapidly recruited via the instantaneously re-established vascular inflow, likely compete with neutrophils for limited interstitial niches [27]. By effectively “out-crowding” pro-inflammatory effectors, this expanded monocyte pool fosters a transiently quiescent, tissue-reparative microenvironment.

We hypothesize that these cells may exist in a functionally quiescent or immature state. This is underscored by the striking numerical homeostasis of the intragraft mature M2 macrophage population (P = 0.691), the significant expansion or stochastic surges of which is typically associated with clinical rejection [28, 29]. While the stable level of MHC-II and PD-L1 on surface of cDC2s suggests that cDC2 could maintain tissue stability through a “low-signal” phenotype [30], literature further indicates that downregulated costimulatory molecules (e.g., CD40, CD80, and CD86) or reduced CCR7 expression typically restricts cDC2 migration and limits “Signal 2” to T cells [31, 32]. Furthermore, specific subpopulation (such as IFNAR1^hi^ TNFR2^+^ cDC2s, iR2D2) are known to be potent sources of IL-10 and IFN-β, which can foster pulmonary homeostasis by inducing regulatory T cells (Tregs), modulating T-helper cell polarization, or mediating antigen-specific T-cell hyporesponsiveness [8, 33]. Within our WOFIE framework, the expansion of this phenotypically stable high-volume cDC2 pool and the arrested differentiation of M2-like monocytes into mature macrophages likely functions as a “local buffer” that prevents early inflammatory signals from escalating into adaptive rejection on POD 7, effectively buying time before the subsequent lymphatic reconnection.

While pulmonary vasculature is restored immediately during surgery, the transection of lymphatic vessels creates a temporary state of “spatial immune decoupling” by precluding the migration of activated APCs to mediastinal draining lymph nodes (dLNs). This anatomical barrier initially favors the DSBT effect because donor antigens in DSBT circulate hematogenously prior to surgery, they preferentially home to the spleen and dLNs [34]. It establishes a regulatory baseline through sub-clinical desensitization. Following LTx, the paralysis of lymphatic efflux reinforces this protection by physically trapping mature APCs within the graft and preventing them from cascade amplifying immune response in dLNs. However, as lymphangiogenesis typically restores connections by POD 14, a “dual anatomical-molecular floodgate” is opened [35, 36]. The synergy of physical reconnection and the molecular upregulation of CCR7 on the backlog of intragraft APCs triggers a synchronized surge into the dLNs. This concentrated influx eventually overwhelms the DSBT-mediated desensitization, leading to the fulminant adaptive response observed on POD 35.

The uniqueness of the lung’s immunological trajectory becomes particularly evident when evaluated against the homeostatic frameworks of other solid organs. Unlike the liver, which possesses an intrinsic filtration and tolerogenic system via Kupffer cells and LSECs rich in immunoregulatory cytokines like IL-10 and TGF-β [37, 38], or the intestine, which utilizes localized gut-associated lymphoid tissue (GALT) networks for *in situ* regulation [39], the lung relies heavily on extrinsic regulation via dLNs [40, 41]. This underscores why the reconnection of lymphatic vessels abruptly shut WOFIE in the lung unlike successful experience in the liver or intestine transplant. Furthermore, the decay of protection on POD 35 may reflect the rapid turnover kinetics of the systemic myeloid reservoir, where the initial DSBT-conditioned regulatory niche is eventually replaced by a secondary wave of recipient monocytes derived from *de novo* hematopoiesis, primed by persistent graft inflammatory signals.

Several limitations warrant consideration. First, the small sample size (n = 5–6) reflects the inherent technical complexity of the murine orthotopic LTx model. Second, while the 24-h preconditioning window presents logistical challenges, it remains manageable via hypothermic preservation strategy [42]. Third, future lineage-tracing and intravital microscopy are needed to definitively confirm the MDP origin and migration kinetics of the observed cDC2s. Furthermore, experiments involving a third-party (non-donor) mouse strain are necessary to confirm whether the observed protective effect is strictly donor-specific. Finally, the failure to induce permanent tolerance—as achieved in other organ models—highlights the need for further optimization of DSBT parameters (e.g., timing, adjunct immunosuppression, and duration) specifically tailored to the lung’s unique immunological environment.

In summary, our findings represent the first successful establishment of a DSBT preconditioning protocol in a murine orthotopic LTx model. We demonstrate that this strategy safely and effectively induces a transfusion-related early protective effect, characterized by preserved graft function and a recalibrated myeloid landscape during the critical first postoperative week. Our hypothesis-generating work establishes a critical baseline for the refinement of DSBT protocols, aiming to achieve sustained graft protection and minimize the requirement for long-term immunosuppression. The predictable nature of this 7-day temporal window provides a foundational platform and a novel translational framework for managing the high immunogenic hurdles inherent to clinical LTx.

## Data Availability

The original contributions presented in the study are included in the article/Supplementary Material, further inquiries can be directed to the corresponding author.

## Supplementary Material

SUPPLEMENTARY FIGURE S1 Gating strategy for flow cytometric analysis of immune cells in the left lung graft and recipient spleen on POD7 after lung transplantation (n=5). Donor- or recipient-derived cells were separated via MHC-I (H2K^d^/H2K^b^) on cell surface. Because the alveolar macrophage is only in the lung graft, the gate of lung interstitial macrophage is the same for all macrophages in the spleen. cDC: conventional dendritic cell; pDC: plasmacytoid dendritic cell; ILC: innate lymphoid cell; LTx: lung transplantation; MHC: major histocompatibility complex; NK: natural killer cell; Treg: regulatory T cell.

SUPPLEMENTARY FIGURE S2 Flow cytometric analysis of donor-derived cells in left lung graft and recipient spleen on POD7 after lung transplantation (n=5). No significant deviation between the control and DSBT group was observed by POD 7. Donor-derived immune cells in the **(A)** left lung graft and **(B)** spleen. *Donor- or recipient-derived alveolar macrophages are excluded from the study because these cells are autofluorescent, which were gated before separating circulating/tissue cells and BALB/c/C57BL/6N); **Cell population for median absolute count <50 is excluded from the study due to interference from the background noise. cDC: conventional dendritic cell; pDC: plasmacytoid dendritic cell; LTx: lung transplantation; Treg: regulatory T cell.
