# Supplementary figures and images for: Donor-Specific Blood Transfusion Induces a Transfusion-Related Early Protective Effect in Murine Lung Transplantation
Source: Transpl Int. 2026 Apr 30;39:15409. doi: 10.3389/ti.2026.15409 (PMC13171468; doi:10.3389/ti.2026.15409)

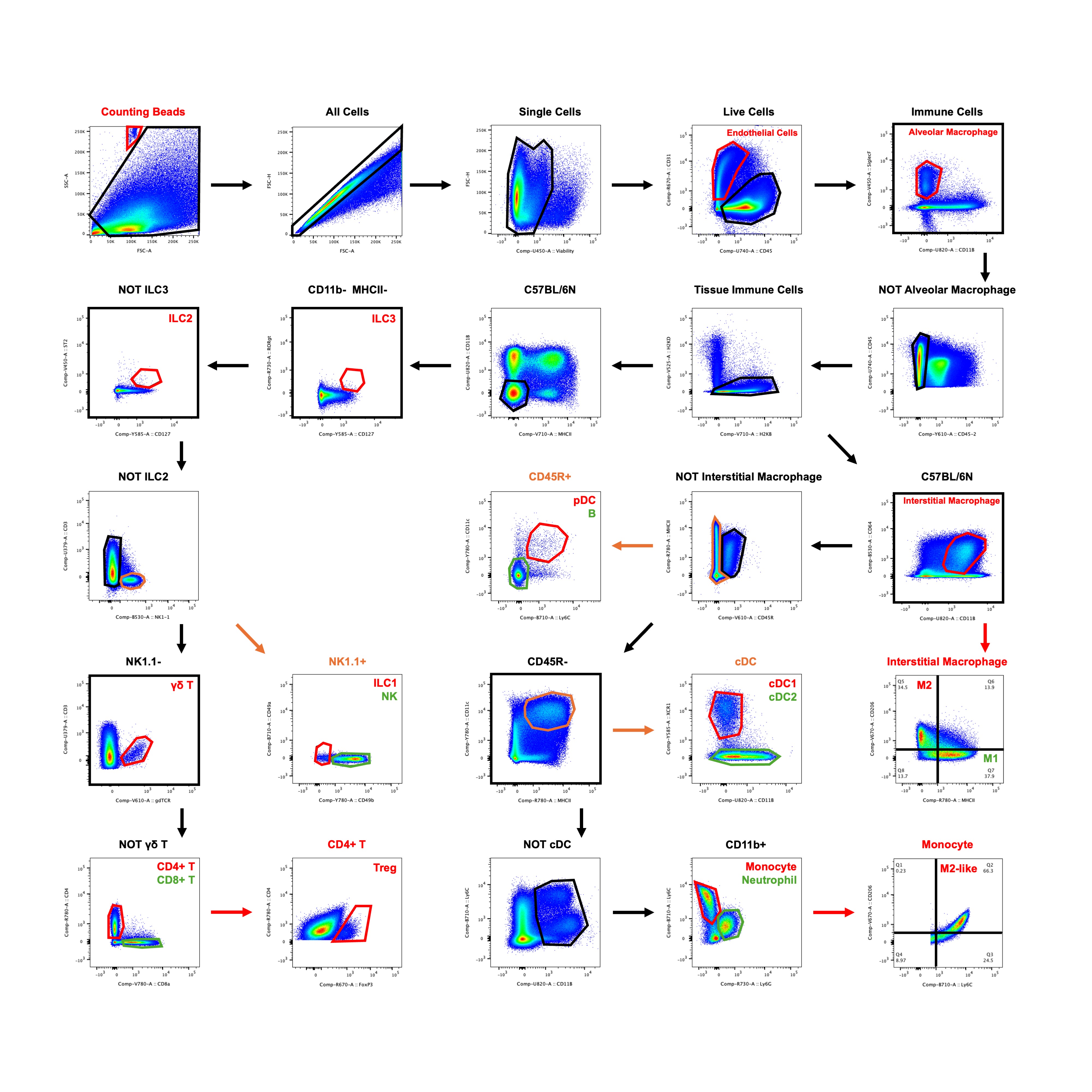

Supplement: Supplementary file 1 [file Image1.jpeg]

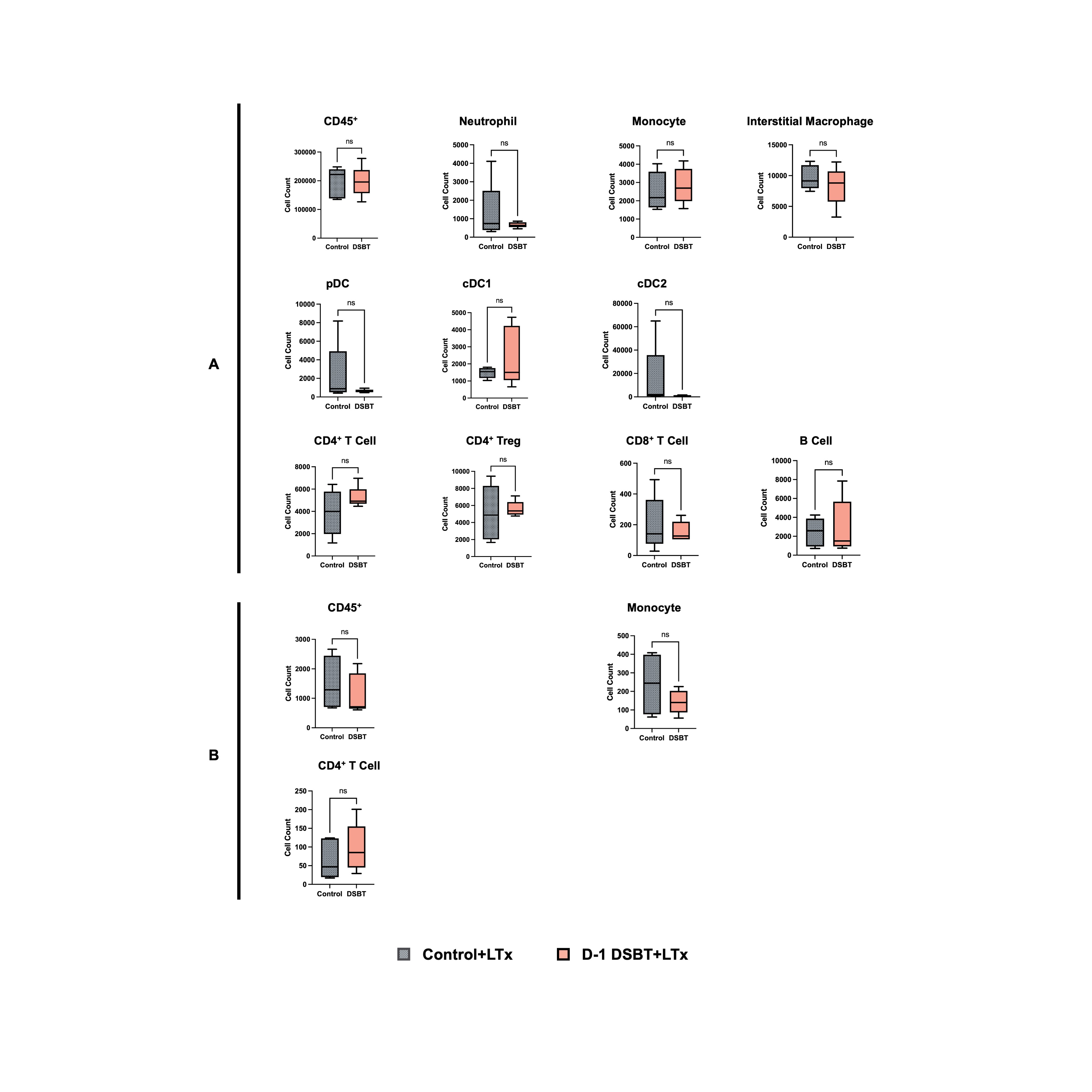

Supplement: Supplementary file 2 [file Image2.jpeg]
